# Inequities in cancer outcomes

**DOI:** 10.2471/BLT.23.290661

**Published:** 2023-09-01

**Authors:** Mohamed El Amine Youcef Ali, Wilma Nusselder, Elisabete Weiderpass, Marilys Corbex, Freddie Bray, Salvatore Vaccarella

**Affiliations:** a Cancer Surveillance Branch, International Agency for Research on Cancer, World Health Organization, 25 avenue Tony Garnier, 69007 Lyon, France.; b Erasmus MC, Rotterdam, Kingdom of the Netherlands.; c Office of the Director, International Agency for Research on Cancer, World Health Organization, Lyon, France.; d Department of Noncommunicable Diseases, World Health Organization Regional Office for Europe, Copenhagen, Denmark.

Socioeconomic determinants have emerged as critical factors in explaining poor cancer outcomes and inequalities among cancer patients, both between and within countries.^1^ A recent study reports that around one third of male and one sixth of female cancer deaths in Europe are associated with a lower socioeconomic position, but this proportion can increase up to half and one fourth, respectively, in former Soviet countries in the Baltic and Eastern Europe.^2^ Indeed, differences in cancer mortality between countries are largely explained by the marked geographic heterogeneity in socioeconomic inequalities.^2^ The situation may well be similar or worse outside Europe, particularly in countries without universal health coverage (UHC), though the magnitude of inequalities in cancer outcomes within countries are not yet measured in most parts of the world, including in many high-income settings.

Socioeconomic inequalities increasingly affect women, as documented by the marked increase in female cancer mortality recorded in the last decades among socioeconomically disadvantaged women, even in countries with a solid tradition of welfare and egalitarian policies, such as the Nordic countries.^2^ These cancer inequalities are not expected to improve in the short-term, given that cancer is already disproportionally affecting more women than men among younger cohorts. As an example, two thirds of all cancers worldwide at ages 20–49 years occur among women, with breast, thyroid and cervical cancer the largest contributors.^3^ While social and economic factors shape cancer outcomes, cancer diagnoses and deaths can have major societal and economic consequences. For instance, deaths due to cancer among young women (aged 15–54 years) are estimated to lead to around 1 million maternal orphans worldwide,^4^ perpetuating the cycle of inequity.

Such results highlight the increasing bearing of socioeconomic and gender inequalities on individuals and societies, and the challenges to attaining Health for All. Therefore, the World Health Organization’s (WHO) WHO Council on the Economics of Health for All Manifesto,^5^ released in 2021, is both timely and welcome. The manifesto transcends the original principle of health being a fundamental human right, emphasizing that health should be of the highest possible standard and accessible to everyone.^5^ The report places health and well-being at the heart of how purpose, value and development are understood, and considers financing for health an investment rather than an expenditure. The manifesto reiterates the essential role of UHC, while recognizing the need to engage with economic and financial leaders; it also establishes that a healthy population must be the ultimate goal of economic activity.^5^

Yet primary investments, and focus on surveillance and research and preventive strategies to reduce these inequalities, are still insufficient. Cancer research funding is far from aligned to the level of cancer burden and the needs related to cancer inequalities,^6^ and studies and intervention measures are rarely designed to narrow the inequalities gap.^7^

Data collection methods that ensure the comparability of harmonized and standardized data across populations are vital to identify particular social groups in specific countries and regions that are at higher risk of developing or dying from cancer. Sufficient investments can serve to inform evidence-based targeted interventions, ensure optimal allocation of resources and enable equitable and accessible high-quality cancer care. For example, the European Cancer Inequalities Registry,^8^ a flagship initiative of Europe’s Beating Cancer Plan,^9^ is certainly a good starting point to measure and tackle cancer inequalities in Europe. The experience of the coronavirus disease 2019 (COVID-19) pandemic has demonstrated how important it is for countries to invest in health systems by addressing the social determinants of health and existing health inequalities.

Policy-makers and political leaders should recognize and prioritize socioeconomic inequities in cancer as a global public health issue, especially in settings where social and economic disparities are widening. Evidence shows that cancer outcomes are strongly affected by socioeconomic factors.^7^ Therefore, advancements and breakthroughs in preventive and curative cancer interventions must be accessible to all.
